# *In situ* Rb-Sr dating of slickenfibres in deep crystalline basement faults

**DOI:** 10.1038/s41598-019-57262-5

**Published:** 2020-01-17

**Authors:** Mikael Tillberg, Henrik Drake, Thomas Zack, Ellen Kooijman, Martin J. Whitehouse, Mats E. Åström

**Affiliations:** 1 Department of Biology and Environmental Science, Linnaeus University, 39231 Kalmar, Sweden; 2 Department of Earth Sciences, Gothenburg University, 40530 Gothenburg, Sweden; 3 Department of Geosciences, Swedish Museum of Natural History, 10405 Stockholm, Sweden

**Keywords:** Geology, Geochemistry

## Abstract

Establishing temporal constraints of faulting is of importance for tectonic and seismicity reconstructions and predictions. Conventional fault dating techniques commonly use bulk samples of syn-kinematic illite and other K-bearing minerals in fault gouges, which results in mixed ages of repeatedly reactivated faults as well as grain-size dependent age variations. Here we present a new approach to resolve fault reactivation histories by applying high-spatial resolution Rb-Sr dating to fine-grained mineral slickenfibres in faults occurring in Paleoproterozoic crystalline rocks. Slickenfibre illite and/or K-feldspar together with co-genetic calcite and/or albite were targeted with 50 µm laser ablation triple quadrupole inductively coupled plasma mass spectrometry analyses (LA-ICP-MS/MS). The ages obtained disclose slickenfibre growth at several occasions spanning over 1 billion years, from at least 1527 Ma to 349 ± 9 Ma. The timing of these growth phases and the associated structural orientation information of the kinematic indicators on the fracture surfaces are linked to far-field tectonic events, including the Caledonian orogeny. Our approach links faulting to individual regional deformation events by minimizing age mixing through micro-scale analysis of individual grains and narrow crystal zones in common fault mineral assemblages.

## Introduction

Dating of faults is of importance for the understanding of faulting histories, local and regional tectonic evolution, as well as mechanisms of faulting and stress release. In cratons, reconstruction of plate tectonics and stress field variations caused by far-field effects of distant orogenic events is aided by geochronological constraints of fault movement. These timing constraints are particularly well-established when combined with kinematic indicators such as the steps in the synkinematic mineral growth that indicate the sense of movement along the fault plane. These minerals outline slickenfibres along the slickenline direction of movement on the slickenside surface where the faulting occurs^1–5^. Geochronological constraints of low-temperature fault evolution can be obtained using the K-Ar and ^40^Ar/^39^Ar techniques for K-bearing minerals and U-Pb and U-Th for carbonates.

For U-Pb, the use of both isotope dilution on multi-grain bulk samples^6–8^ and high spatial resolution spot analysis within grains^9–11^ have been shown to be suitable for dating calcite crystals in faults. High spatial resolution techniques are well-suited in crystalline rock fractures as they allow dating of different generations of mineral overgrowths and can be focused on tiny mineral overgrowths on primary minerals of the host rock^9^. However, very low U concentrations, and, in particular, open system behavior for U, have inhibited previous *in situ* dating attempt of slickenfibre calcite in crystalline rocks of the Baltic Shield^10^. The multi-grain bulk sample methods on the other hand, although able to succeed with lower U concentrations, face difficulties due to the fine-grained nature of minerals on fault surfaces. In addition, the tiny zonations within fault minerals and overgrowths of secondary minerals on primary wall rock mineral nuclei result in mixed ages of several mineral growth generations.

Bulk sample K-Ar and ^40^Ar/^39^Ar techniques are widely used for geochronological determination of illite formation in faults^12–14^, but feature difficulties of grain-size dependence on the usually large range of obtained ages, as well as problems with argon diffusion and mixing of ages from several generations of mineral growth in the bulk data^15,16^. Recent developments in multi grain-size K-Ar analysis of fault gouge illite have enabled temporal distinction of grain-size controlled authigenic growth generations from detrital components, thus revealing multiple reactivation events in a complex set of faults, samples or grain sizes through establishing a model that makes use of the inclined age spectra of repeatedly reactivated faults^2,5,17–19^. In comparison to these innovations in characterizing fault histories by bulk sample analysis, the development of analytical techniques for detailed detection of discrete overgrowths or recrystallization within individual mineral grains has not yet reached similar applicability. A few *in situ* ^40^Ar/^39^Ar studies applying laser probe spot sizes down to 70 μm on fault zones^20,21^ have provided initial indications that geologically significant high-precision *in situ* dating has the potential to increase knowledge of microscale mechanisms and systematics involved in faulting while overcoming the challenges of inheritance, mixing and isotopic closure associated with current dating techniques of slickenfibre precipitates in faults.

Here we present application of the newly developed *in situ* Rb-Sr dating method^22,23^ to fine-grained slickenfibre mineral coatings on sub-surface faults in several deep cored boreholes in Paleoproterozoic metagranites. The high spatial resolution (50 μm spot size) dating was performed by using laser ablation triple quadrupole inductively coupled plasma mass spectrometry analyses (LA-ICP-MS/MS) on K-feldspar, illite, calcite and albite crystals on the outermost edges of stepped, slickensided fault surfaces. Stable O isotope composition in calcite was determined, via Secondary Ion Mass Spectrometry (SIMS), to further ensure dating of a single mineral growth event. The slickenfibre nature of the dated minerals makes it possible to link the mineral growth to existing kinematic information in the form of mineral steps on the fracture surfaces^24,25^. In this first report of *in situ* Rb-Sr dating of fine-grained mineral precipitates (slickenfibres) in fault zones, we present and evaluate a protocol for *in situ* Rb-Sr geochronology of fault minerals and apply it to a sample set that gives a framework for the tectonic reconstruction within a Proterozoic craton.

## Geological Setting and Investigated Deformation Zones

**Figure 1:**
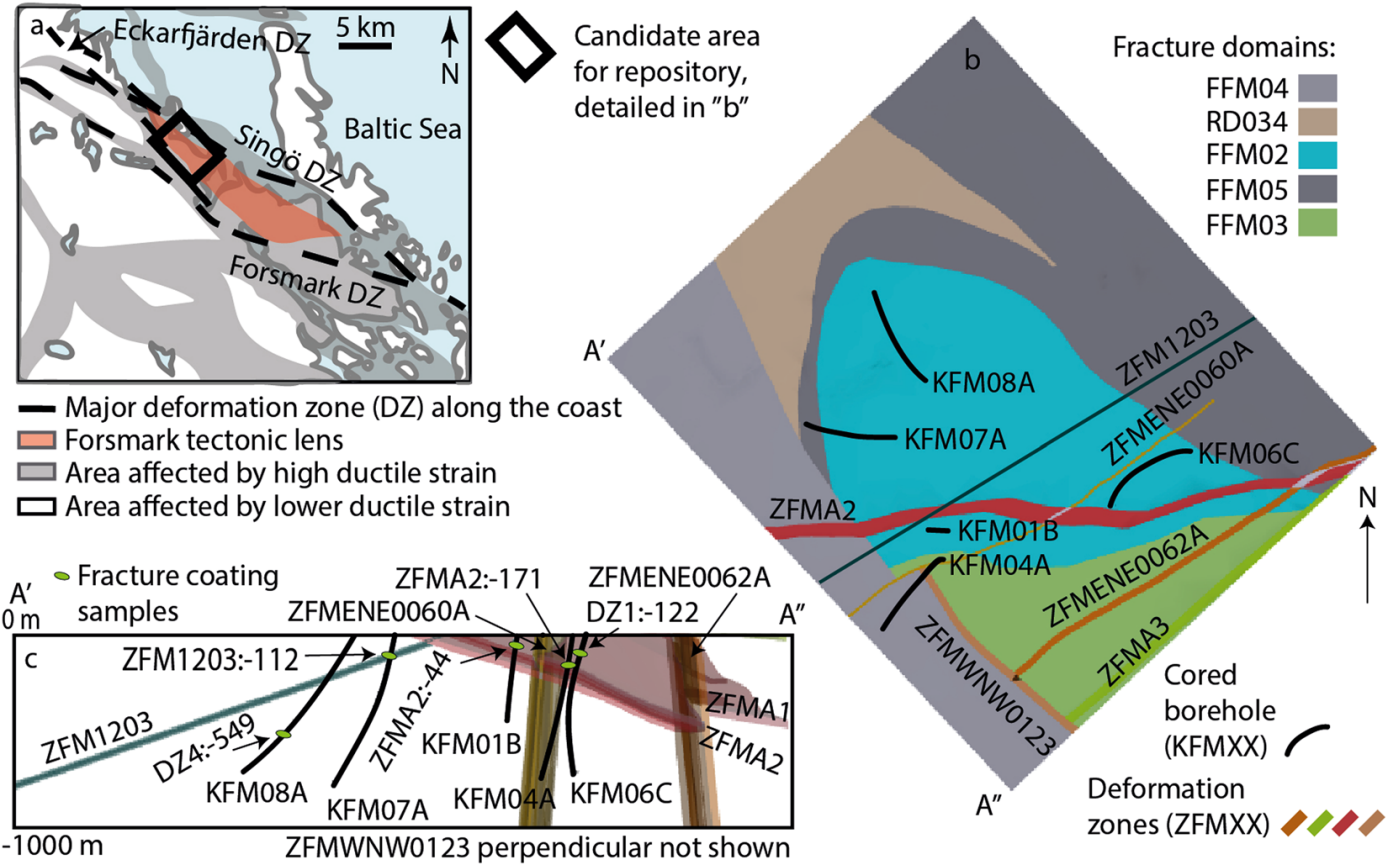
Maps of geological features. (**a**) Regional Forsmark area with ductile strain character, surrounding major shear zones and Forsmark tectonic lens indicated. (**b**) Surface map of local Forsmark area (rectangle in “a”) with color coding simply to distinguish the different fracture domains (see Olofsson *et al*.^70^ for details), deformation zones and sampled boreholes (with sub-surface projection). (**c**) Profile view of transect A’-A” in (**b**) showing the sub-surface orientation of the deformation zones and the location of the mineral samples at the borehole intercept of the zones. The samples in KFM08A and KFM06C intersect possible deformation zones that are not modeled stochastically and thus not existing in the 3D database. Modified from Stephens *et al*.^27^, and based on data extracted from the site database (Sicada).

The plutonic rocks in the study area (Forsmark, Sweden) formed during the Svekokarelian orogeny with crystallization ages typically in the 1.89 to 1.87 Ga range^26^. The two dominant groups of rocks are both felsic: (1) Fine- to medium-grained granodiorite, tonalite and subordinate granite and (2) Biotite-bearing granite (to granodiorite)^27^. The rocks were affected by penetrative ductile strain under amphibolite-facies metamorphic conditions from 1.87 to 1.86 Ga when ductile high-strain belts with WNW-ESE to NW-SE trend developed^25,28^. These shear zones anastomose around tectonic lenses with an inferred lower degree of ductile strain (Fig. 1a). One of the lenses is termed the Forsmark tectonic lens, and is the target area for a deep geological repository for spent nuclear fuel in Sweden^27^, therefore being a geological body of first order societal relevance. The deformation regime shifted to semi-brittle and brittle at 1.8 to 1.7 Ga^29^. Paleostress analysis, cross-cutting relations of various mineral coating generations and some ^40^Ar/^39^Ar dating^25,27,30^ have been used to construct a model of the brittle evolution of the area. According to Saintot *et al*.^25^, the brittle evolution started with transpressive deformation with a regional NNW-SSE trend, and clockwise stress deviation within the tectonic lens. This resulted in dextral slip along steep WNW-ESE and NW-SE deformation zones, at ca. 1.8 Ga. A NE-SW directed transpressive strain is inferred to have been active at 1.7–1.6 Ga. Later, 1.1–0.9 Ga transpressive WNW-ESE σ_1_ deformation in response to the Sveconorwegian orogeny to the west resulted in sinistral reactivation along the WNW-ESE and NW-SE zones. Reactivation of any of the aforementioned brittle structures during even younger Phanerozoic tectonic events could not be excluded, but has not been identified in previous kinematic investigations^25^. Extensional paleostress fields are also evident in the geological record at Forsmark, with Svecokarelian and Sveconorwegian transtensive regimes due to stress permutations, and to regional extensional tectonic regimes during later regional tectonics events. The latter include reactivation at 456 ± 2 Ma of Proterozoic NNE-SSW- and ENE-WSW-oriented fractures due to an early Caledonian orogenic event^30^ and Permian reactivation at 277 ± 1 Ma of Proterozoic NNE-SSW-, ENE-WSW- and NW-SE-oriented fractures due to regional rifting^30^. Moreover, mineral precipitation in open fractures occurred during the Devonian-Carboniferous late- and post-Caledonian collapse at 402 ± 9.4 Ma and 355 ± 14 Ma^9^, and in the Jurassic (173 ± 7.6 Ma)^9^.

The gently dipping and W-striking major deformation zone ZFMA2 (Fig. 1b,c) was investigated in two borehole intercepts, named ZFMA2:-44 and ZFMA2:-171 after the deformation zone and the sample depth (in meter) relative to sea level that basically corresponds to the ground surface (Fig. 1c). The host lithology is a strongly foliated, medium-grained metagranite with minor pegmatitic granite, amphibolite and fine-grained metagranitoid. Several depth intervals display strongly increased frequency of open, commonly gently dipping fractures. In the core of the deformation zone where ZFMA2:-44 was sampled, steep N-S strike-slip faults occur in a section with abundant sub-horizontal fractures^25^. The ZFMA2:-171 section of the zone mostly features gently dipping, open fractures and fracture frequencies of open and sealed fractures in the 10–20 /m range^31^.

The gently dipping and WSW-striking deformation zone ZFM1203 (Fig. 1b,c) intersected over 75 m borehole length and consists of a concentration of open fractures dipping gently to the NNW and a few crush zones. It was sampled at –112 m (ZMF1203: -112). The zone has intervals of elevated fracture frequencies classified as fault core separated by longer intervals where fractures are much less prevalent^32^.

The DZ1:-122 sample is from a minor deformation zone with drill core intervals of increased fracture intensity and/or crushed rock at 102–169 m borehole length in metagranite with pegmatite and some amphibolite. The DZ4:-549 sample is from a possible deformation zone occurring over 21 m borehole length in medium- to coarse-grained metagranite with some amphibolite and pegmatite. The zone has a fracture frequency of approximately 6 fractures/m.

## Results

**Figure 2:**
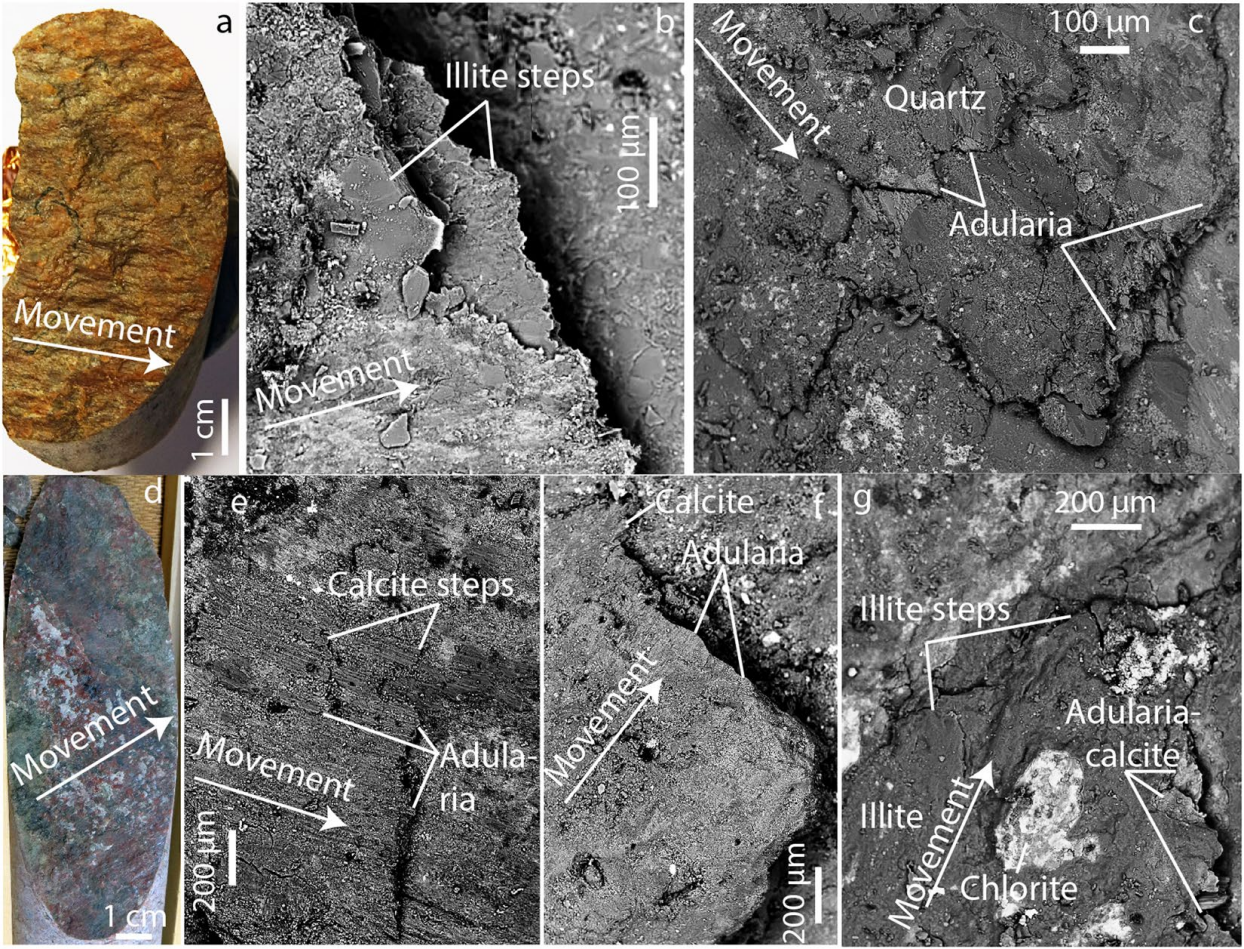
Fracture surfaces (**a,d**, photographs) and micro-textures (**b,c**, **e–g** back-scattered SEM-images showing representative detailed examples of common features throughout the fracture surfaces). (**a–c**) Samples that yielded Proterozoic ages, and (**d–g**) samples that yielded Paleozoic ages. (**a**) Photograph of the slickensided open fracture surface of sample DZ4:-549. (**b**) Illite at the outermost tips of a stepped slickensided fracture surface, sample DZ4:-549. (**c**) Adularia grown at the outermost tips of steps of a slickensided fault surface in sample ZFM1203:-112. (**d**) Sample DZ1:-122, with sinistral movement indicated by calcite steps and lineation on illite (light green), chlorite (dark green) and hematite-stained adularia (red). (**e**) BSE-SEM image of the fracture surface in (**d**) showing slickensided fabric and stepped appearance of fault surface with adularia and calcite grown at the outermost tips. (**f**) Slickensided fabric and stepped surfaces with adularia and calcite grown at the outermost tips, sample ZFMA2:-171. (**g**) Slickensided fabric and stepped surfaces with illite, adularia and calcite grown at the outermost tips, sample ZFMA2:-44. Movement of the missing blocks over the fracture surfaces is indicated, as interpreted in this study based on the stepped appearance of the fracture surface morphology (slickenfibres) and the orientation of the slickenlines. The relative motion of the fracture surface is hence in the opposite direction of the arrow.

Sample photographs and microphotographs are provided in Fig. 2 and in Supplementary Fig. S1, whereas Supplementary Dataset S1 and Supplementary Dataset S2 provide the sample and reference data for LA-ICP-MS/MS Rb-Sr analysis and MC-LA-ICP-MS ^87^Sr/^86^Sr analysis, respectively.

**Figure 3:**
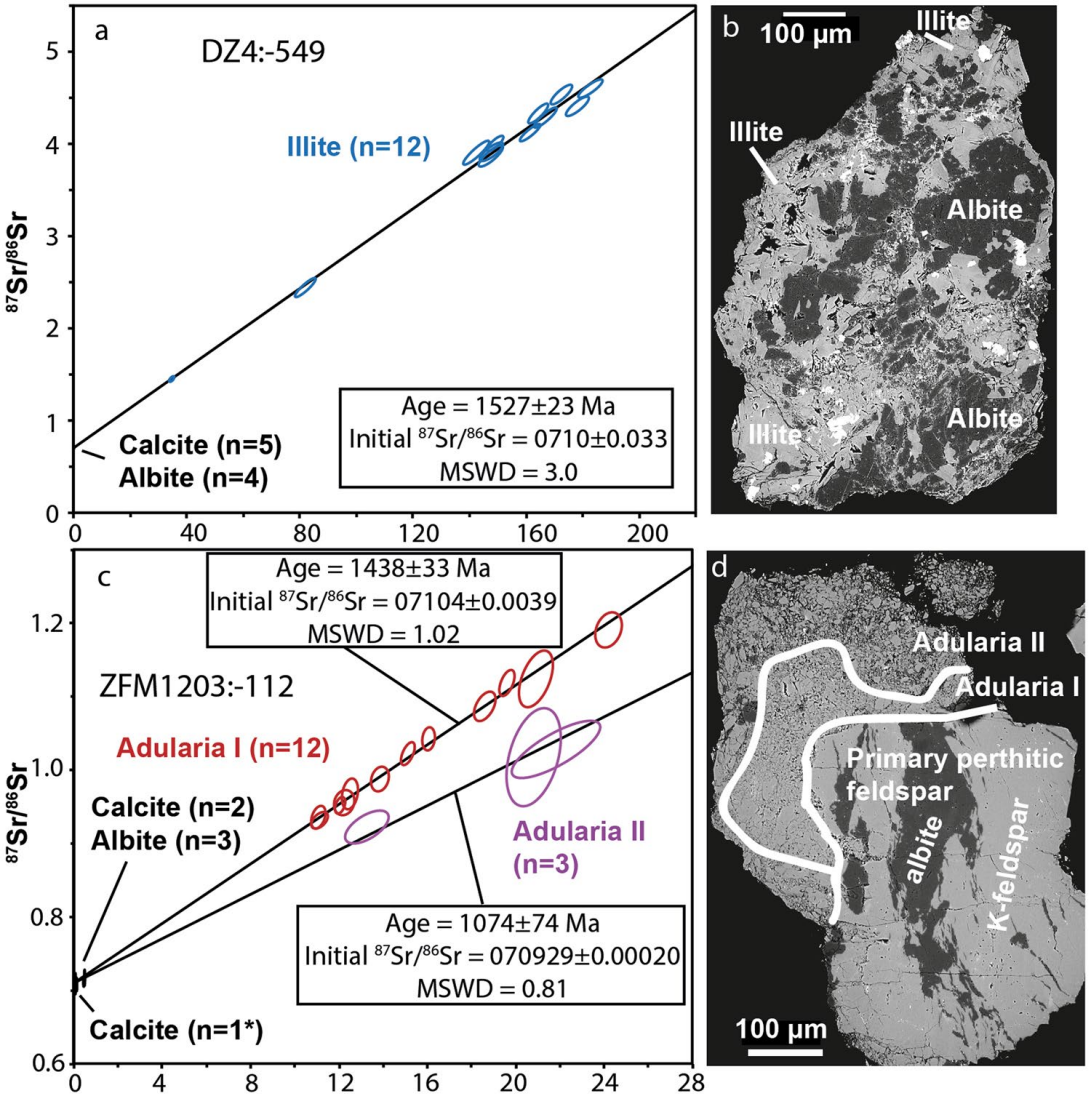
Rb-Sr isochrons and corresponding examples of Proterozoic mineral assemblages used for LA-ICP-MS spot analysis. (**a**) Sample DZ4:-549, yielding 1527 ± 23 Ma, (**b**) slickenfibre mineral assemblage, consisting of albite and illite. (**c**) Sample ZFM1203:-112, yielding two isochrons at 1438 ± 33 Ma and a more uncertain at 1074 ± 74 Ma where the initial ^87^Sr/^86^Sr value is derived from Sandström and Tullborg^57^, (**d**) fine-grained adularia (with small amounts of albite = dark) overgrowths of two generations on primary perthitic wall rock feldspar on the slickensided surfaces on the open fracture. Errors represented by the ellipses are relative standard errors based on 1σ standard deviations.

The fracture surface of DZ4:-549 has a slickensided smooth appearance due to frictional movement between the footwall and the hanging wall. There are irregularities in the fault plane, with exposed slickenfibres that have a stepped appearance showing a reverse (hanging wall up) sense of movement across the fault (Fig. 2a, Supplementary Fig. S1k). The mineral assemblage of the outermost slickenfibres was dominated by illite (Fig. 2b) with intergrown albite (Fig. 3b) and some calcite. A Rb-Sr isochron constructed by 12 illite microanalyses and an initial ^87^Sr/^86^Sr composition determined by analyses of albite (n = 4) and calcite (n = 5) yielded an age of 1527 ± 23 Ma (MSWD = 3.0, Fig. 3a).

The fracture where sample ZFM1203:-112 was taken displays growth of adularia, albite and calcite on the slickensided surface, and at the edges of stepped fault surfaces (Fig. 2c, Supplementary Fig. S1i). The fine-grained adularia-dominated secondary assemblage was clearly distinguishable from perthitic primary feldspar in the polished aliquots used for microanalysis (Fig. 3d). In the assemblage there were two different growth zone generations (Fig. 3d) yielding two age populations with Rb-Sr isochrons (Fig. 3c): 1438 ± 33 Ma (MSWD = 1.02; *n*_*adularia*_ = 12), and a less well-constrained 1074 ± 74 Ma due to fewer analytical spots of the Rb-rich mineral adularia (MSWD = 0.81; *n*_*adularia*_ = 3). It is not straightforward to distinguish separate slickenfibres representing the two different fault generations detected in the geochronological data. Since the first generation of secondary adularia is found in the same stepped areas of the slickensided fracture surface as the younger overgrowths, a similar sense of shear for both events is likely.

**Figure 4:**
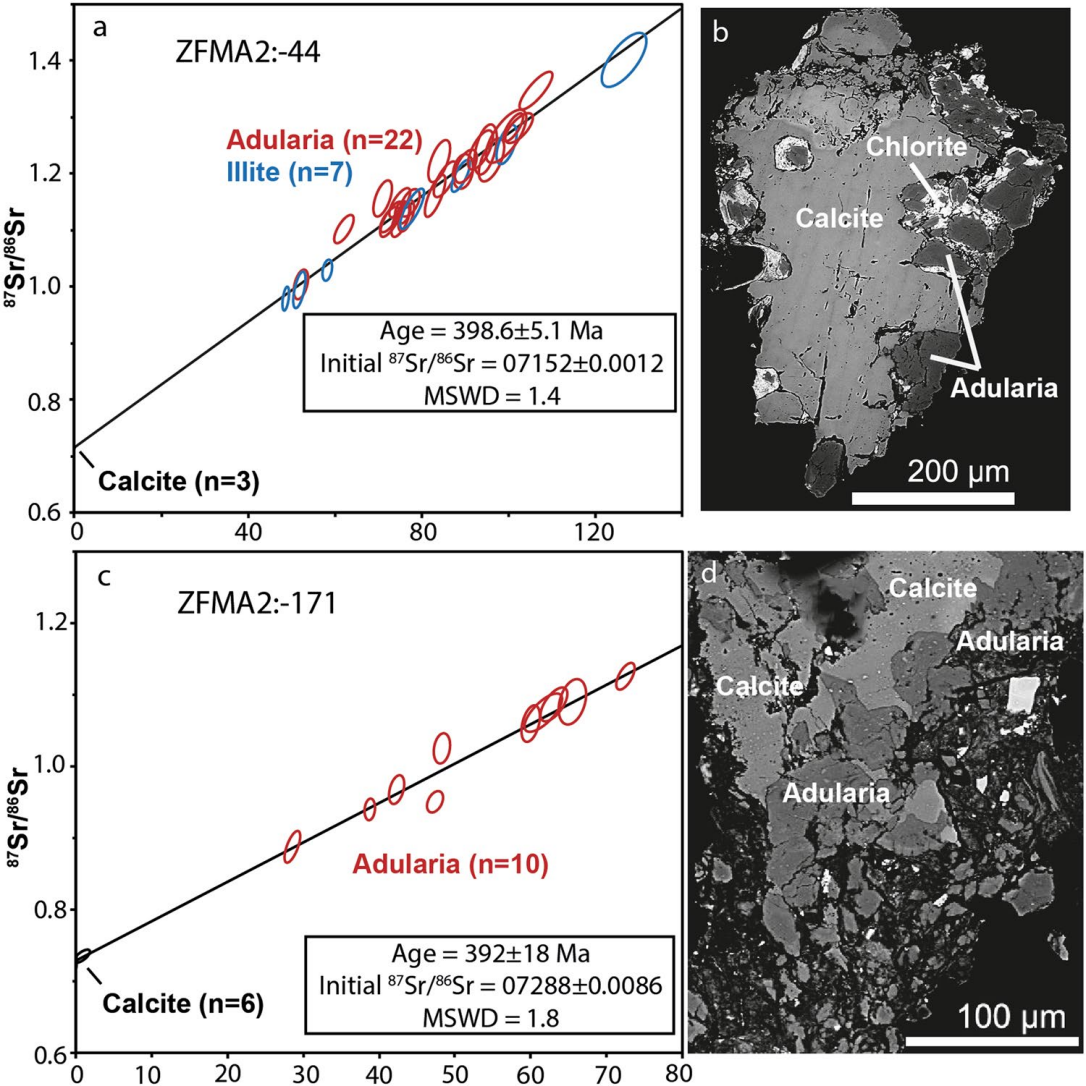
Rb-Sr isochrons and corresponding examples of Devonian mineral assemblages used for LA-ICP-MS spot analysis (**a**) Sample ZFMA2:-44, yielding 398.6 ± 5.1 Ma, (**b**) slickenfibre mineral assemblage consisting of calcite, chlorite and adularia (illite existing in other aliquots). (**c**) Sample ZFMA2:-171m, yielding 392 ± 18 Ma, (**d**) slickenfibre mineral assemblage consisting of fine-grained intergrown calcite and adularia. Errors represented by the ellipses are relative standard errors based on 1σ standard deviations.

Sample ZFMA2:-44 is from a N-S-oriented fault in the deformation zone core decorated by adularia, illite and calcite as observed with SEM (Fig. 2g, Supplementary Fig. S1c). This mineral assemblage occurred at the outermost tips at steps on the slickensided fracture surface. An isochron constructed by microanalyses of illite (n = 7) and adularia (n = 22) together with calcite for initial ^87^Sr/^86^Sr in the fine-grained intergrown mineral assemblage (Fig. 4b) yielded an age of 399 ± 5 Ma (MSWD = 1.4, Fig. 4a). In the same deformation zone, ZFMA2:-171 has a mineral assemblage of adularia and calcite (Fig. 2f, Supplementary Fig. S1f) with intergrown texture (Fig. 4d) at the outermost tips at steps on the slickensided fracture surface. An isochron constructed by mineral-specific microanalyses of adularia (n = 10) and calcite (n = 6) yielded an age of 392 ± 18 Ma (MSWD = 1.8, Fig. 4c).

**Figure 5:**
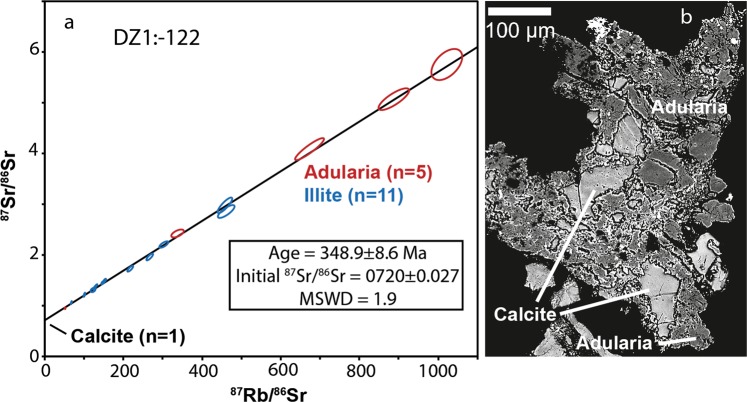
Rb-Sr isochrons and corresponding examples of Carboniferous mineral assemblages used for LA-ICP-MS spot analysis (**a**) Sample DZ1:-122, yielding 348.9 ± 8.6 Ma, (**b**) slickenfibre mineral assemblage consisting of fine-grained intergrown calcite and adularia. Errors represented by the ellipses are relative standard errors based on 1σ standard deviations.

The sample DZ1:-122 is a fault with calcite steps, illite, chlorite and hematite-stained adularia (Fig. 2d, Supplementary Fig. S1i,n). Detailed SEM investigations reveal that adularia and calcite (Fig. 2e) occur together with illite on the outermost tips of the stepped, slickensided fault plane, and that these minerals are intergrown (Fig. 5b). An isochron constructed of adularia (n = 5), illite (n = 11) and calcite (for initial ^87^Sr/^86^Sr) yielded an age of 349 ± 9 Ma (Fig. 5a, MSWD = 1.9).

## Discussion

**Figure 6:**
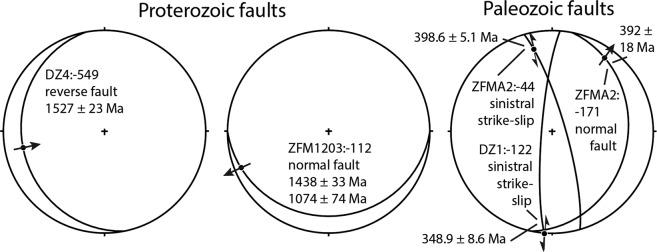
Stereoplots of fault slip data derived from Saintot *et al*.^25^ with fault planes as lines and striae as arrows according to indicated movement and sense.

**Figure 7:**
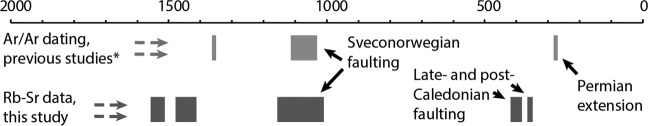
Timeline in millions of years before present showing the distribution of previous fracture mineral geochronological data (*=^30,71^) compared with the data in this study.

Here we link the radiometric information derived from microanalyses of the mineral assemblages (slickenfibres) in faults to kinematic data from the same fracture surfaces to distinguish episodes of fault activation. We relate the activation events to well-established temporally constrained deformation phases in the far-field to understand block movement within a fragmented craton in general and to determine fault activation within the tectonic lens of the Forsmark area, in particular (Figs. 6 and 7). First, detailed appraisal of the geochronological results is compared and related to specific faulting mechanisms and the regional thermal history. After this, an evaluation of the application of the *in situ* Rb-Sr methodology to fault dating is discussed in terms of technical and geological requirements, as well as possibilities. The δ^18^O calcite data indicative of the source of fluids present at the point of precipitation is provided in Supplementary Dataset S3 and discussed in Supplementary Note.

### Linking faulting to tectonic events

Two fracture mineral slickenfibre assemblages yielded Proterozoic ages (Figs. 3a,c). The 1527 ± 23 Ma reverse dip-slip in DZ4:-549 is consistent with maximum compressive stress (σ_1_) oriented WSW-ENE. This is roughly in line with NE-SW bulk crustal shortening during the Gothian orogeny affecting southwestern Sweden at 1.75–1.55 Ga^33,34^. However, in eastern Sweden and western Finland this period is commonly ascribed to NE-SW extensional tectonics^1,25^, which disagrees with the reverse dip-slip in DZ4:-549. This discrepancy may be due to local stress modifications within the tectonic lens fault architecture. The host rocks cooled below c. 300 °C at 1.73–1.66 Ga as inferred from biotite ^40^Ar/^39^Ar ages^29^ and below 200–225 °C at 1.55–1.49 Ga as inferred from K-feldspar ^40^Ar/^39^Ar ages of the host rocks^35^ and from U-Th/He thermochronology further south in the Fennoscandian craton^36^. If our Rb-Sr ages represent cooling below the closure temperature at which the Rb-Sr decay system in illite is not reset or disturbed, or if repeated faulting caused complete resetting^37^, the movement represented by the slickenfibres should be even older than the age we obtained. Temperature- and/or fluid-induced recrystallization/diffusion may reset both the Rb-Sr^38,39^ and argon isotope systems^3,4^ at substantially lower temperatures than those typically used for isotopic closure. However, short hydrothermal or frictional heating events are not expected to affect illite ages even above 250 °C^5^. Since the Rb-Sr system has been shown to be more resistant to resetting than radiogenic ^40^Ar^40^, the slickenfibre illite likely recorded the timing of faulting.

The Rb-Sr ages of the two different adularia populations in the assemblages of the gently southward dipping, WSW-striking, ZFM1203:-112 (Figs. 3c and 6) both coincide with known tectonic events. The 1438 ± 33 Ma age is similar to ^40^Ar/^39^Ar muscovite ages found further south (Laxemar area, SE Sweden) of altered wall rocks adjacent to veins and greisen related to adjacent Mesoproterozoic A-type granites^41^ associated with the Danopolonian-Hallandian orogenies, which in the south featured WSW-ENE-directed crustal shortening^42^. If the Rb-Sr closure temperature of adularia is similar to that for ^40^Ar/^39^Ar (150 ± 30 °C^43^;), the regional temperature regime of >200–225 °C^35,36^ recorded in the Paleo-Mesoproterozoic of the Fennoscandian shield may have been high enough to reset grains grown at an earlier stage. This adularia age can thus be regarded as a minimum age of fault propagation. The less well-constrained younger Rb-Sr age (1074 ± 74 Ma) is in broad agreement with the Sveconorwegian orogeny affecting western Scandinavia at 1.1–0.9 Ga^44^ with WNW-ESE to mainly E-W crustal shortening^45^ and similarly oriented transpression in southern Sweden^25,46,47^, and western Finland^1,48^, which agrees relatively well with the strike/dip and strike-slip striae orientations. The obtained Rb-Sr age also overlaps early Sveconorwegian adularia- and laumontite-sealed breccia ^40^Ar/^39^Ar ages of ~1.1 Ga in the Forsmark area^30,49^. Thermal history modeling indicates low temperatures at ~1.1 Ga, but also later Sveconorwegian reheating between 944 Ma and 851 Ma at an average temperature of ~220 °C^36^. The 1074 ± 74 Ma age is consequently proposed to represent precipitation during faulting and not isotope disturbance by later reheating.

Paleozoic Rb-Sr ages are recorded in three samples (Figs. 4a,c and 5a). The borehole intercepts of the gently dipping major deformation zone ZFMA2 feature similarly aged mineral assemblages of 399 ± 5 Ma (ZFMA:-44) and 392 ± 18 Ma (ZFMA2:-171). Sinistral sense of shear affecting the N-S-oriented steep strike-slip fault of ZFMA2:-44 indicates a σ_1_ from NW, which is in line with NW-SE to WNW-ESE Caledonide bulk crustal shortening^25,33^. The striation of ZFMA2:-171 is subparallel to the orientation of the same deformation zone hosting ZFMA2:-44 and thus also roughly agrees with the Caledonian bulk crustal shortening, although slightly more NNW-trending than commonly ascribed to maximum compressional stress during the Caledonian orogeny. The stress axis during Paleozoic re-activation in the ZFMA2 zone may, however, also be influenced by local transpression within the tectonic lens that is squeezed in between the major bordering shear zones. The observed striations may otherwise be an indication of an early episode of the 0.4–0.25 Ga Variscan orogeny^50^ on the European continent that remains poorly constrained in Fennoscandia^23^. Temperature constraints below 100 °C in the basement rocks and fracture coatings at Forsmark at this time^9,36,51^, along with coinciding illite and adularia ages and the absence of textural and isotopic disturbance or fluid-induced recrystallization in the grains, confirm that the Paleozoic ages obtained are slickenfibre formation ages.

In the possible deformation zone DZ1, the fault with calcite-illite-adularia dated to 349 ± 9 Ma is a N-S steep strike slip fault with sinistral sense of shear. Similar to the steep fracture in the ZMFA2 zone, the N-S steep strike slip DZ1:-122 fault with sinistral sense of shear indicates that the σ_1_ roughly oriented NNW-SSE agrees with Caledonide bulk crustal shortening^25,33^. However, the 349 ± 9 Ma age is younger than the bulk crustal shortening episode and in a phase when extension prevailed in the Caledonides^52–54^. For this deformation zone, Variscan crustal shortening in the south may be a more plausible origin for the observed lineation and slickenfibres.

Geochronological constraints of even younger tectonic events such as 95 to 60 Ma inversion tectonics with dextral transpressional deformation along the Sorgenfrei-Tornquist Zone^55^ or development of far-field Alpine 65–55 Ma conjugate strike-slip faults in the platform limestones at Öland^8^, were not detected.

### Evaluation of method feasibility

The high level of detail on both spatial and depth scales by the new *in situ* fault dating technique emphasizes a close assessment of the significance of and relation between individual textural, chemical and geochronological features, following the procedure that in our recent study proved decisive for successful linkage of hydrothermal veins to granitic magmatism^56^. In that study, *in situ* Rb-Sr dating of muscovite/biotite ± K-feldspar ± calcite ± fluorite ± epidote in greisen and veins emanating from a Mesoproterozoic granite yielded ages (1432 ± 8 Ma) in accordance with crystallization age of the granite (1433 ± 10 Ma), thus confirming the temporal connection between granite, greisen and veins and a correspondence of the U-Pb (zircon) and the *in situ* Rb-Sr geochronological approaches. In the light of the new *in situ* Rb-Sr dating application presented here for low temperature fault minerals, the validity and applicability of the method on the actual fault movements are concretized by assessing how data is produced and interpreted, and in what way critical isotopic and kinetic requirements are met by the analytical method.

#### Isochron construction

An isochron is constructed from multiple spots with variance in both internal and compared ratios of ^87^Rb/^86^Sr and ^87^Sr/^86^Sr. In our samples, at least two sample spots (usually many more) each of two different mineral phases constitute an isochron, which requires isotopic homogeneity and equilibrium on the scales of single spots and zones or grains, as well as amongst each mineral and co-genetic assemblage. Homogeneity within each spot is obtained by ensuring stability throughout the laser ablation signal and by using a common standard error for the isotopic ratios for every spot. The signal stability corresponds to the laser crater descent depth on a <1 µm scale and provides not only information on the radiogenic isotopic distribution, but also on other chemical heterogeneities such as zonation, inclusions, alterations or inheritance. Detection of these heterogeneities is crucial as they typically induce offset in the isotopic Rb-Sr ratios that must remain intact for valid isochrons. Although the distributions and concentrations of the radiogenic isotopes may remain in equilibrium even if they are disturbed by contamination, heterogeneities have consistently been screened off to ensure that the isochron age of the individual spot constitutes a representation of the timing of isotopic closure. The relatively tedious data reduction procedure thus results in rejection of a significant part of the analyzed spots during quality control. In this specific study, 83 out of the total of 185 spots were rejected due to heterogeneities or high ratio errors (Supplementary Dataset S1). Analytically reliable signals of a zone or grain generation are subsequently grouped into assemblages according both to their texturally and structurally established co-existence, as further described below, and by isochron model fitting that monitors closed system conditions and discerns any trace of inconsistent inheritance or mixing of distinct fault events. Isochron ages, errors and other statistical measures (Supplementary Dataset S1) are used for comparison between assemblages and generations in and amongst fault sets.

#### Controlling isotopic equilibrium and homogeneity

The assumption of isotopic equilibrium between each spot and mineral contained in the isochron is foremost controlled by petrographic analysis to determine the paragenetic relationship between coeval mineral phases. The careful separation of minerals and fragments from slickensides after initial SEM-characterization is followed by crystal-specific SEM-examination of cross-sections of the epoxy-mounted grain used for dating, in order to detect chemical heterogeneities. In closely associated adularia-calcite and adularia-albite slickenfibre pairs (Figs. 3d, 4d and 5b), complex crystal zonation formed from episodic growth is tackled through the microscale analytical capability to target each individual zone. The growth zones can texturally and temporally be recorded from the oldest generation formed onto the wall-rock to the outermost precipitation corresponding to the latest fault reactivation event. Furthermore, initial ^87^Sr/^86^Sr values of calcite are compared with the extensive database on calcite-filled fractures in the Forsmark area^57,58^. Intergrown co-genetic phases occur also with illite (Fig. 3b), but petrographic recognition of illite generations in spot-size scale is inhibited by the fine grain size of reactivated illite and instead relies upon geochemical homogeneity recorded downhole during laser ablation. Compositional homogeneity obtained by the time-resolved laser ablation signal plots is thus crucial, not only in the illite spots but also in the accompanying Sr-rich mineral. In addition to the aforementioned textural and radiogenic isotopic control, stable isotope compositions of calcite also provide control of that the outermost minerals represent the same faulting events. Furthermore, the microscale nature of the analytical spots infers that dating is dependent on the selectivity of illite fragments considering that the illite grain size reduction during repeated fault reactivation causes grain size-dependent age inclination. Extensive spatial sampling and analytical campaigns are therefore recommended to detect all reactivation events that caused illite neocrystallization. Single spots are unlikely to incorporate all reactivation events, unlike bulk analysis which will constitute a mix of all generations in a sample size large enough. The *in situ* method on the other hand distinguishes and controls the distribution of generations both in a spatial and depth notion. This level of detail can also detect the influence of isotope resetting, independently on whether neocrystallization of illite occurred or not during fault propagation or fluid pervasion. A mixed age spectrum can be sorted out both on the scale of individual spots and of an assemblage of zones or grains. In order to obtain readable age data, a specific zone or an assemblage of submicrometer-sized grains must be thick and homogenous enough to obtain a steady laser ablation signal for tens of micrometers.

#### Isotopic and mineral-specific sources of errors

The phase mixing of illite with other clay minerals, often smectite, which commonly forms by transformation and authigenesis is not recorded directly by the method on the scale of fine multi-reactivated clay grains. Distinction of illite polytypes into muscovite or smectite varieties also requires other methods than geochronological analysis. Each signal interval of a dated illite spot will therefore not be characterized mineralogically. However, irregularities in the chemical distribution of single spots and assemblages are recognized through element variation with and amongst LA signal lengths. Since individual generations have proven to be distinguishable both chemically and age-wise with the *in situ* dating method aided by petrographic observations, fraction separation is not required for temporally constraining the reactivation history of faults.

Occasionally determined broad age errors yielding imprecise constraints of faulting can have several analytical causes, including the 1 sigma age error to account for internal errors, high errors of ^87^Rb/^86^Sr and ^87^Sr/^86^Sr as well as low ^87^Rb/^86^Sr / ^87^Sr/^86^Sr of the Rb-rich mineral(/s), and high ^87^Sr/^86^Sr error of the Sr-rich mineral. Fault reactivation with short time spans in between is a geological factor that would increase the isochron age error if undistinguishable by the structural, textural, and chemical analyses. Mineral mixing or other chemical impurities are, however, distinguishable factors that can be avoided. Although errors are not propagated from secondary standard samples, dating of these ensures method validity in the absence of application of previously dated faults.

#### Applicability and outlook

The presence of calcite in all dated samples offers initial Sr fix points in the isochrons and an indication of a fluid-rich environment during fault slip rather than long-term seismic creep as the governing precipitation mechanism^8^. Furthermore, the zonation displayed by adularia and calcite indicates that nucleation of crystals results in growth as recrystallization possibly preceded by partial dissolution along crystal boundaries. By connecting fluid properties and behavior with fault mechanisms, the integrated approach using textures, structures, dating and stable isotope characterization capacitates insight into fluid-rock interactions during brittle deformation.

The *in situ* Rb-Sr method is indeed useful for discriminating faulting events on a time resolution of millions of years, but, alike other geochronological analytical tools, remains inaccurate in detecting the temporal history of multiple reactivation events close in time within each fault event. This reinforces the importance of structural and textural analysis of the fault coating samples. A comprehensive study spread laterally and vertically across a particular fault surface that has yielded comparable geochronological constraints by another isotope system would further explore the applicability and validity of *in situ* Rb-Sr dating of faulting. The combination of grain size separation and diffraction of illite fault gouge could provide valuable understanding on the Rb-Sr systematics in the dimension provided by laser ablation analysis.

Taken together, the *in situ* method can be regarded as complementary to Ar-based bulk analysis since the latter has no possibility to target individual zones or grains. The clear textural, chemical and temporal distinction of synkinematic mineral formation highlights the potential of our *in situ* Rb-Sr fault dating protocol to resolve multi-stage brittle deformation of bedrocks and thus for regional paleo-tectonic reconstructions.

## Conclusions

The geochronological constraints derived from the minerals formed at the outermost tip of stepped slickensided fracture surfaces represent major events of fault reactivation within the Fennoscandian craton. High spatial resolution microanalyses were crucial to derive temporal information from these fine-grained mineral assemblages, which include tiny overgrowths on primary wall rock minerals and close intergrowth between several co-genetic phases. Assemblages of illite and/or adularia together with co-genetic calcite and albite in sub-horizontal and sub-vertical faults disclose slickenfibre growth at several occasions spanning over 1 billion years from at least 1527 Ma to 349 ± 9 Ma. The ages of these growth phases and the structural orientation information derived from the kinematic indicators on the fracture surfaces can be linked to far-field tectonic events, including the Proterozoic Gothian, Danopolonian-Hallandian, Sveconorwegian and Caledonian orogenies. Integrated textural analysis is central in achieving the clear temporal distinction of synkinematic mineral formation and recrystallization on slickensides. Our protocol ensures geochemical homogeneity and isotopic equilibrium of the geochronological data through rigorous control of isochron requirements on the individual spot scale within discrete grain zonations of the fault gouge mineral assemblages. The capacity to distinguish generations of micro-scale mineral growth enables linkage of complex fault reactivation sequences to local deformation events and craton-scale tectonism.

## Methods

### Sampling

In the Forsmark area, the Swedish Nuclear Fuel and Waste Management Co (SKB) has performed site investigations for a spent nuclear fuel repository deep within the metagranitic bedrock^59^. During these investigations, several cored boreholes have been drilled with the triple-tube technique^60^, which is excellent for preservation of fragile slickenfibre precipitates, because the core pieces are not rotated within the tube during drilling (Fig. 2a). Orientation data for strike/dip of each fault plane has been retrieved through post-drilling downhole camera documentation^61^. Sampling was focused to open fractures in deformation zones, and in particular to fractures that were included in previous extensive kinematic studies^24,25,62,63^. In the present study we have re-examined the fracture surfaces with SEM-EDS, which was not the case in the earlier studies. This allowed collection of more detailed mineralogical information/identification than previous routine mapping [e.g.^64^] and the above mentioned kinematic studies.

### Sample materials

One drill core sample from each of the five deep boreholes was targeted. The dated samples were from four, dominantly sub-horizontal deformation zones in the Forsmark area. Zones ZFMA2 and ZFM1203 are of regional character and are intercepted by the boreholes KFM01B, (sample ZFMA2:-44), KFM04A (ZFMA2:-171) and KFM07A (ZFM1203:-112, Fig. 1), and two minor deformation zones (DZ1:-122, and DZ4:-549).

### Characterization

The fault surfaces were first characterized in a Hitachi S-3400N scanning electron microscope (SEM) at Earth Sciences Centre, University of Gothenburg, Sweden. The SEM was equipped with an Oxford Instruments energy dispersive spectrometer (EDS) used to identify the mineral assemblages through semi-quantitative microanalysis, by linking the energy to oxide standard measurements and drift-correction to a co-mounted cobalt standard. Specifically, K-and Ca-rich mineral precipitates suitable for Rb-Sr dating were located in the outermost tips of stepped surfaces of slickensided fault surfaces.

### Mounting

Grains of K-feldspar (adularia), albite, illite and calcite located by SEM characterization in the outermost tips of stepped surfaces were hand-picked from the fracture surface under a microscope. The grains were embedded in epoxy mounts, which were polished to expose crystal centers. The cross sections were investigated with SEM as above to distinguish secondary mineral assemblages from primary nuclei (Fig. 2d, showing fine-grained secondary adularia on top of perthitic feldspar) and in a few cases also more than one generation of secondary mineral precipitation (Fig. 2d, showing two phases of secondary adularia growth). These SEM observations were used to guide the LA-ICP-MS analysis by documenting areas within the grains that were large enough for the LA spot, and free from inclusions and micro-cracks.

### Rb-Sr analysis

The Rb-Sr dating system is based on the beta-decay of ^87^Rb to ^87^Sr in minerals. One or several Rb-rich minerals (showing increased ^87^Sr/^86^Sr and decreased ^87^Rb/^86^Sr with time) along with a co-genetic Sr-rich mineral (constant ^87^Sr/^86^Sr with time), in our case adularia and/or illite in paragenesis with calcite and/or albite (mineral relations in Figs. 2, 3, 4 and 5), were analysed by Rb-Sr geochronology with 50 μm spot size via a newly developed high spatial resolution LA-ICP-MS method^23^ at the University of Gothenburg, Sweden utilizing a 213 nm NWR laser ablation system coupled to an Agilent 8800QQQ ICP-MS. Separation of ^87^Rb from ^87^Sr is achieved by producing oxide of ^87^Sr ions as the ablated material is ionized in the plasma of the ICP-MS and reacts with N_2_O^22^ in a reaction cell positioned between two quadrupoles. For each analytical session, reference materials were selected to ensure that the pulse/analog setting of each measured isotope ratio was identical in samples and reference materials. The ^87^Rb/^86^Sr and ^87^Sr/^86^Sr calibration of the raw ratios of samples is performed using session-based means from repeated analysis of glass standards BCR-2G and NIST SRM 610 or 612. Over the course of three analytical sessions, BCR-2G had ^87^Rb/^86^Sr within-run precisions of 0.85–1.39%, whereas BCR-2G, NIST SRM 610 and 612 had within-run precisions of 0.32–0.48, 0.25% and 0.62%, respectively. Mica-Mg, a pressed nanopowder pellet of a phlogopite separate with an established age of 519.4 ± 6.5 Ma^22^ constituted a secondary reference material for two analytical sessions, yielding 518 ± 27 Ma (n = 31 (n_rejected_ = 1); mean square weighted deviation (MSWD) = 5.4) and 519 ± 22 Ma (n = 26; MSWD = 3.7). For one session, LP01, a sample comprising mm-sized euhedral biotite from granodiorite of the La Posta intrusion in California served as a secondary standard, yielding an isochron age (92.0 ± 2.6 Ma; n = 10 (n_rejected_ = 4); MSWD = 0.37) overlapping with the literature weighted mean age of 91.6 ± 1.2 Ma^23^. The resulting standard and sample ages are isochron model fits constructed using the recently updated Rb decay constant^65^. Secondary standard isochrons along with all sample and standard data are provided in Supplementary Dataset S1. Rho (ρ) values for each spot were calculated using the 1 s errors of ^85^Rb/^86^Sr, ^87^Sr/^86^Sr and ^85^Rb/^87^Sr (^85^Rb is used as a proxy for ^87^Rb as it is constant on Earth and within 0.02–0.05%^66^). Average count rate calculation of standard data is conducted by Glitter^©^, whereas sample data reduction and within-run error calculation of element and isotope ratios is performed using an in-house spreadsheet. Individual spot analyses for Rb-rich minerals were screened and those below 5% error for ^87^Rb/^86^Sr and ^87^Sr/^86^Sr and below 10% for the relative standard error of the ratio between ^87^Rb/^86^Sr and ^87^Sr/^86^Sr were used, with an additional criterion of at least ten seconds of steady ablation (Supplementary Dataset S1). No error propagation from uncertainties in literature data or within-run errors of standards is applied to sample errors, because internally calculated errors are significantly larger than the established 1.5% external errors of this method^22^. The ^87^Sr/^86^Sr values of calcite from two of the dated samples were established by micro-scale MC-LA-ICP-MS analysis of the growth zone within the calcites that was in paragenesis with adularia. These analyses were carried out using a Nu plasma (II) MC-ICP-MS at the Vegacenter, Swedish Museum of National History, Stockholm. Laser ablation was done using an ESI NWR193 ArF excimer laser ablation system. The ^87^Sr/^86^Sr values are corrected for possible interferences including Rb, doubly-charged rare earth elements, Kr and Ca-dimers and –argides. To avoid interference from (Ca/Ar)^31^P^16^O^+^, oxide levels are monitored by scanning Th/ThO and are kept < 0.5%. After interference correction, ^87^Sr/^86^Sr values are corrected for linear drift and normalized to an inhouse brachiopod reference material *Ecnomiosa gerda*. A modern oyster shell from Western Australia has been run as secondary reference material, which is compared to the ^87^Sr/^86^Sr of modern seawater. Values of the reference measurements and the analytical settings of the MC-LA-ICP-MS analytical sessions are given in Supplementary Dataset S2.

### Oxygen isotope analysis

Slickenfibre calcite was mounted in epoxy, polished and analysed for ^18^O/^16^O with Secondary Ion Mass Spectrometry (SIMS) on a Cameca IMS1280 ion microprobe in spots of 10 μm lateral beam dimension, 1–2 μm depth dimension. Two to three analyses were made within each crystal. In total 19 analyses were made in calcite for δ^18^O in calcite from samples ZFMA2:-44, ZFMA2:-171, DZ4:-549. C and O isotope already existed for DZ1:-122^9^. Settings follow those described previously^67–69^. Spots were placed in areas in the crystals without micro-fractures or inclusions. Calcite results are reported as per mil (‰) δ^18^O based on the Pee Dee Belemnite (V-PDB)-standard value. Analytical session was carried out running blocks of six unknowns bracketed by two reference material analyses (full data in Supplementary Dataset S3). Isotope data from calcite were normalised using calcite reference material S0161, from a granulite facies marble in the Adirondack Mountains, kindly provided by R.A. Stern (Univ. of Alberta). The values used for IMF correction were determined by conventional stable isotope mass spectrometry at Stockholm University on ten separate pieces, yielding δ^18^O:-5.62 ± 0.11‰ V-PDB (1 std. dev.) Precision was δ^18^O: ± 0.2–0.3‰. Reference material measurements are listed in Supplementary Dataset S3.

## Supplementary information

Supplementary Note.

Supplementary Figure S1.

Supplementary Dataset S1.

Supplementary Dataset S2.

Supplementary Dataset S3.

## Data Availability

All relevant data are included in the Supplementary material to this article.Supplementary datasets are Supplementary Dataset S1: Rb-Sr data for samples and reference materials, Supplementary Dataset S2: ^87^Sr/^86^Sr values of samples and reference materials, Supplementary Dataset S3: O isotope values of calcite samples and reference material, Supplementary Fig. S1: Microphotographs of fracture sample appearance and textures, and Supplementary Note: Discussion on O isotope values of calcite samples.
