# Supplementary material for: In situ Rb-Sr dating of slickenfibres in deep crystalline basement faults
Source: Sci Rep. 2020 Jan 17;10:562. doi: 10.1038/s41598-019-57262-5 (PMC6969261; doi:10.1038/s41598-019-57262-5)
Supplement: Supplementary file 1 — Supplementary Note. [file 41598_2019_57262_MOESM1_ESM.docx]

**­Supplementary Information to**

**In situ Rb-Sr dating of slickenfibres in deep crystalline basement faults**

**Mikael Tillberg^a,b^*, Henrik Drake^a^, Thomas Zack^b^, Ellen Kooijman^c^, Martin J. Whitehouse^c^, Mats E. Åström^a^**

^a^Department of Biology and Environmental Science, Linnaeus University, 39231 Kalmar, Sweden
^b^Department of Earth Sciences, Gothenburg University, 40530 Gothenburg, Sweden
^c^Department of Geosciences, Swedish Museum of Natural History, 10405 Stockholm, Sweden

*Corresponding author. E-mail address: mikael.tillberg@lnu.se

Supplementary note

δ^18^O calcite data is provided in Dataset S3. Here, the significance of these values is discussed. The oxygen isotope signatures of calcite have previously been shown to partly reflect wall-rock influence but mainly the groundwater composition and hydrochemical reactions driven by infiltration, circulation and mixing of different water types^1^. The calcite of the 1527±23 Ma old DZ4:-549 sample has a relatively homogeneous δ^18^O composition of -13.7±0.3‰ V-PDB (n=9), which is in line with previous values of fracture calcite precipitated during this period^2,3^.

The calcite in the two fractures with Paleozoic mineral assemblage ages; 399±5 Ma (ZFMA:-44) and 392±18 Ma (ZFMA2:-171) has relatively homogeneous δ^18^O compositions of -11.0±0.8‰ (n=7, ZFMA2:-44) and -14.7±0.4‰ (n=3, ZFMA2:-171m). This homogeneous O isotope composition suggests that the calcite was formed during a single event, particularly because the O isotope composition in calcite has varied significantly over time in the fracture system^2,3^.

The calcite crystals in the 349±9 Ma old mineral assemblage in DZ1:-122 are larger and better developed (euhedral) than on the other fracture surfaces, and the previously reported δ^18^O and δ^13^C composition in the calcite indicate two phases of precipitation; an early formed slickenfibre with δ^18^O of -6.3±2.5‰ and δ^13^C of -7.2±1.6‰ that is intergrown with adularia, and a later phase with δ^18^O of -10±0.8‰ and δ^13^C +7.2±1.9‰ that reflects low-temperature *in situ* microbial methanogenesis through carbonate reduction pathways in the fracture system^2^. The latter process is interpreted to post-date the frictional movement at 349±9 Ma, but may be related to documented Jurassic extensional fracture reactivation and associated microbial activity in the fracture system^2^.

Supplementary References

1 Drake, H., Heim, C., Hogmalm, K. J., & Hansen, B. T. Fracture zone-scale variation of trace elements and stable isotopes in calcite in a crystalline rock setting. *Applied Geochemistry*, **40**, 11-24 (2014).

2 Drake, H. *et al.* Isotopic evidence for microbial production and consumption of methane in the upper continental crust throughout the Phanerozoic eon. *Earth and Planetary Science Letters* **470**, 108-118 (2017).

3 Sandström, B. & Tullborg, E.-L. Episodic fluid migration in the Fennoscandian Shield recorded by stable isotopes, rare earth elements and fluid inclusions in fracture minerals at Forsmark, Sweden. *Chemical Geology* **266**, 126-142 (2009).
