# Supplementary material for: In situ Rb-Sr dating of slickenfibres in deep crystalline basement faults
Source: Sci Rep. 2020 Jan 17;10:562. doi: 10.1038/s41598-019-57262-5 (PMC6969261; doi:10.1038/s41598-019-57262-5)
Supplement: Supplementary file 2 — Supplementary Figure S1. [file 41598_2019_57262_MOESM2_ESM.docx]

**­­Supplementary information to:**

**In situ Rb-Sr dating of slickenfibres in deep crystalline basement faults**

**Mikael Tillberg^a,b^*, Henrik Drake^a^, Thomas Zack^b^, Ellen Kooijman^c^, Martin J. Whitehouse^c^, Mats E. Åström^a^**

^a^Department of Biology and Environmental Science, Linnaeus University, 39231 Kalmar, Sweden
^b^Department of Earth Sciences, Gothenburg University, 40530 Gothenburg, Sweden
^c^Department of Geosciences, Swedish Museum of Natural History, 10405 Stockholm, Sweden

*Corresponding author. E-mail address: mikael.tillberg@lnu.se

Supplementary Figure S1


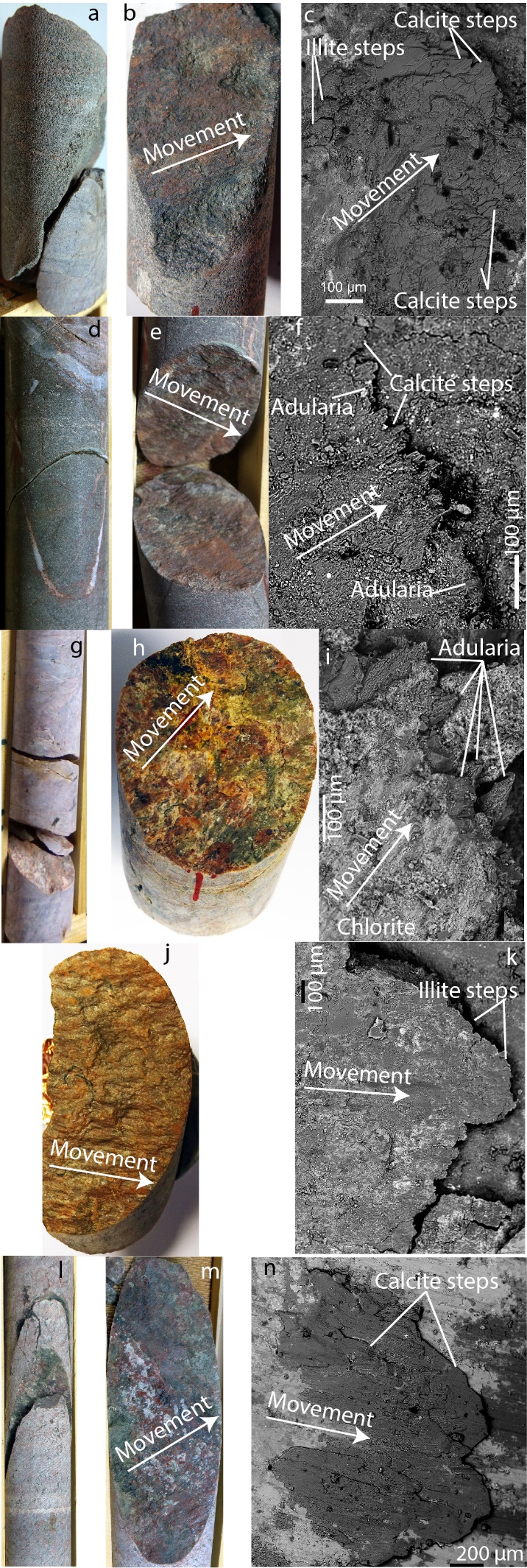


Fig. S1. Microphotographs of fracture sample appearances and textures. Showing columns of (left) photograph of fracture in drill core box, (centre) photograph of fracture surface (DZ4:-549 missing) and (right) SEM image of mineral assemblage at stepped fracture surface. (a-c) ZFMA2:-44, with hematite-stained illite and adularia visible in the photograph, whereas the SEM image shows calcite and illite on the outermost tips of stepped slickensided fault surfaces. (d-f) ZFMA2:-171, showing hematite-stained adularia, chlorite and calcite on the fracture surface photograph and close up of calcite and adularia on in the stepped surfaces on the fault plane. (g-i) ZFM1203:-112 showing bright red adularia and green chlorite/illite, white calcite and albite in the fracture surface photograph and close-up of adularia slickenfibres. (j-k) DZ4:-549 with illite on the outermost stepped edge of the slickensided surface. (l-n) DZ1:-122 with hematite-stained adularia, illite, chlorite and calcite on the fracture surface. SEM close up image shows calcite steps. Drillcore width in (a,b,d,e,g,h,j, l and m) is 5 cm.
